# Lack of efficacy of troglitazone at clinically achievable concentrations, with or without 9-*cis* retinoic acid or cytotoxic agents, for hepatocellular carcinoma cell lines

**DOI:** 10.1038/sj.bjc.6602200

**Published:** 2004-10-05

**Authors:** Y-C Shen, C Hsu, J-Y Chen, A-L Cheng

**Affiliations:** 1 Department of Oncology, National Taiwan University Hospital, Taipei, Taiwan; 2 Department of Internal Medicine, National Taiwan University Hospital, Taipei, Taiwan; 3 Graduate Institute of Clinical Medicine, National Taiwan University College of Medicine, Taipei, Taiwan; 4 Division of Cancer Research, National Health Research Institutes, Taipei, Taiwan

**Keywords:** troglitazone, 9-*cis* retinoic acid, hepatocellular carcinoma, cytotoxic chemotherapy

## Abstract

Although the PPAR*γ* agonist troglitazone has been shown to induce growth inhibition of hepatocellular carcinoma (HCC) cells at high concentration, this study indicates troglitazone does not significantly inhibit the growth of HCC cells at clinically achievable concentrations (1–10 *μ*M), and this lack of activity could not be improved by the addition of 9-*cis*-retinoic acid. Furthermore, no synergistic effect was found between troglitazone and cytotoxic anticancer agents.

The peroxisome proliferator-activated receptor *γ* (PPAR*γ*), a member of the nuclear hormone receptor superfamily, functions as a ligand-dependent transcription factor and plays an important role in several signalling pathways, including lipid metabolism, glucose homeostasis, and inflammation (Kersten *et al*, 2000). Heterodimerisation of PPAR*γ* and retinoid X receptor (RXR) is required for binding to specific DNA response elements of target genes. Binding with either PPAR*γ* or RXR ligands will elicit transcriptional activation of the target genes (Vanecq and Latruffe, 1999; Corton *et al*, 2000). Synergistic activation of downstream genes may occur when both ligands are present (Kliewer *et al*, 1992; Desvergene and Wahli, 1999). The thiazolidinediones were found to be specific agonists of PPAR*γ*, with EC50 less than 1 *μ*M (Lehmann *et al*, 1995). The potency of the thiazolidinediones in activating PPAR*γ in vitro* was found to correlate closely with their lipid- and glucose-lowering activity *in vitro* (Berger *et al*, 1996; Willson *et al*, 2001). Troglitazone, a thiazolidinedione derivative, has been demonstrated to induce adipocyte proliferation and differentiation with the concentrations of 0.5–5 *μ*M (Tafuri, 1996).

The anticancer activity of PPAR*γ* agonists was first demonstrated in a liposarcoma model. Thiazolidinedione derivatives induced terminal differentiation of liposarcoma cells (Tontonoz *et al*, 1997; Demetri *et al*, 1999; Tsibris *et al*, 1999). Subsequent studies indicated that PPAR*γ* agonists, at concentrations of 1–10 *μ*M, may induce growth inhibition in a variety of cancers, including cancers of breast, colon, and prostate (Kubota *et al*, 1998; Sarraf *et al*, 1998; Mehta *et al*, 2000). In addition to promotion of cell differentiation, the PPAR*γ* agonists-induced growth inhibition may involve various mechanisms such as induction of cell cycle arrest, inhibition of DNA synthesis, and increase of cancer cell necrosis and apoptosis. Moreover, synergistic or additive effects of growth inhibition between PPAR*γ* and RXR*α* agonists have been found in liposarcoma and breast cancer cells. Clinical trials exploring the feasibility of using PPAR*γ* agonists in the treatment of human cancers are underway (Koeffler, 2003).

Recent studies have demonstrated that PPAR*γ* agonists may induce growth inhibition in hepatocellular carcinoma (HCC) cells in a dose- and time-dependent manner (Koga *et al*, 2001; Rumi *et al*, 2001; Yoshizawa *et al*, 2002). Troglitazone, a thiazolidinedione derivative, enhanced the expression of the cyclin-dependent kinase inhibitors p21^WAF1/Cip1^ and p27^Kip1^ and resulted in cell cycle arrest at G0/G1 phase. PPAR*γ* agonists may also augment Fas-mediated apoptosis of HCC cells induced by tumour necrosis factor *α* (Okano *et al*, 2002). However, these effects were observed at relatively high concentrations (20–50 *μ*M) of troglitazone, while the clinically achievable concentrations are around 2–5 *μ*M (Berger *et al*, 1996; Tafuri, 1996; Spencer and Markham, 1997; Willson *et al*, 2001). Therefore, the utility of PPAR*γ* agonists for the treatment of HCC remained undetermined.

The current study was designed to address the following questions: (1) whether troglitazone alone, at clinically achievable concentrations, may be active against HCC cells; (2) whether troglitazone has a synergistic effect with RXR*α* agonists on growth inhibition of HCC cells; (3) whether the effects of troglitazone and RXR*α* agonists correlate with the expression of PPAR*γ* and RXR*α* in HCC cells; and (4) whether troglitazone, at clinically achievable concentrations, may enhance the cytotoxic effects of major chemotherapeutic agents.

## MATERIALS AND METHODS

### Cell culture and reagents

A panel of HCC cell lines was tested in this study: Hep3B, HepG2, SNU-449 (Park *et al*, 1995), (purchased from ATCC), PLC-5, Huh-7 (Nakabayashi *et al*, 1982), SK-hep1 (Fogh *et al*, 1977), HA-59T (Wuu *et al*, 1990) (gifts of Professor Ming-Yang Lai, Graduate Institute of Medicine, College of Medicine, National Taiwan University). MCF-7 (purchased from ATCC), a breast cancer cell line characterised by expression of RXR*α* and 9-*cis* retinoic acid (9-*cis* RA) growth inhibition, was used as positive control for RXR*α* expression and 9-*cis*-RA-induced cytotoxicity in this study (Gottardis *et al*, 1996). The HCC cells and MCF-7 were cultured in Dulbecco's modified Eagle's medium supplemented with 10% foetal bovine serum, 100 U/ml penicillin, and 100 *μ*g/ml streptomycin, and maintained in a humidified incubator of 5% CO_2_ in air at 37°C.

Troglitazone was purchased from Cayman Chemical (Ann Arbor, MI, USA) and 9-*cis*-RA was purchased from Sigma (St Louis, MO, USA). We used 9-cis retinoic acid in our study because in earlier studies it has been demonstrated to have synergistic activity with PPAR*γ* agonists in regulation of lipid metabolism and in a leiomyosarcoma model. Both troglitazone and 9-*cis*-RA were prepared in dimethyl suphoxide (DMSO) for cytotoxicity assay. The final concentration of DMSO was controlled at below 0.5%. The cytotoxic agents were provided by the following companies: gemcitabine, Eli Lilly; irinotecan, Rhone–Poulenc–Rorer; cisplatin, David Bull Laboratories; paclitaxel, Bristol-Myers-Squibb. Polyclonal mouse antibodies detecting PPAR*γ* and rabbit antibodies detecting RXR*α* were purchased from Santa Cruz Biotechnology (Santa Cruz, CA, USA). Monoclonal antibody detecting proliferating cell nuclear antigen (PCNA) was purchased from Chemicon (Temecula, CA, USA). The anti-PPAR*γ* antibody we used in this study was mapped to the N-terminal of PPAR*γ* (sc-7196, Santa Cruz, CA, USA). No cross reaction with other PPAR isoforms has been reported by the manufacturer. The dilution of antibodies was 1 : 1000 for primary antibodies and 1 : 2500 for secondary antibodies.

### Extraction of nuclear proteins and Western blot analysis

The methods of nuclear protein extraction and Western blot analysis have been described previously (Chuang *et al*, 2002). Briefly, cells (3–5 × 10^6^) were scraped from culture dishes, washed with iced phosphate-buffered saline, resuspended in 1 ml of buffer A (1.5 mM MgCl_2_, 10 mM KCl, 0.5 mM dithiothreitol, 0.2 mM PMSF, 1% Nonidet P-40, 10 mM HEPES, pH 7.9), and incubated for 10 min at 4°C. The cells were then centrifuged at 1500 rpm for 2 min at 4°C and the pellet was resuspended in 0.1 ml of buffer B (25% glycerol, 420 mM NaCl, 1.5 mM MgCl_2_, 0.2 mM EDTA, 0.5 mM dithiothreitol, 0.2 mM PMSF, 20 mM HEPES, pH 7.9) and incubated for 20 min at 4°C. The cell suspension was then centrifuged at 14 000 rpm for 2 min at 4°C, and the supernatant was collected for protein quantification using a modified Lowry's method. The supernatant was stored in aliquots at −70°C. A measure of 30 *μ*g of protein was deposited in each lane on the blot. The proteins were separated by SDS–PAGE and transferred to nitrocellulose membranes. The membranes were incubated with the appropriate primary antibodies, followed by incubation with horseradish peroxidase-conjugated secondary antibodies and a chemiluminescence agent (Santa Cruz). The proteins were then detected by roentgenography.

For each study at least three independent experiments, including independent cultures, protein collection and quantification, were done and separate blots were done to detect the expression of PPAR*γ* and RXR*α*. The most representative figures were shown.

### Cytotoxicity assay

Cytotoxicity was determined by a tetrazolium-based semiautomated colorimetric assay (MTT assay) (Mosmann, 1983). Three independent experiments of each cytotoxicity test have been done and the results were the mean of the three experiments. For each experiment at least three replicates were done. Cells were plated in 96-well plates 3–4 × 10^3^ well^−1^) and incubated overnight. The drugs were added to the wells and the percentage of surviving cells was measured by MTT assay after continuous drug exposure for 72 h. Concurrent drug exposure was used to evaluate the combination effect of troglitazone with 9-*cis*-RA and troglitazone with four cytotoxic agents (gemcitabine, cisplatin, paclitaxel and irinotecan) that have different mechanisms of cytotoxicity. The IC50 of individual cell lines was calculated by using a linear regression based on the MTT assay results. The drug concentration corresponding to 50% inhibition of control was designated as IC50.

## RESULTS

### Expression of PPAR*γ* and RXR*α* and single-agent activity of troglitazone

**Figure 1:**
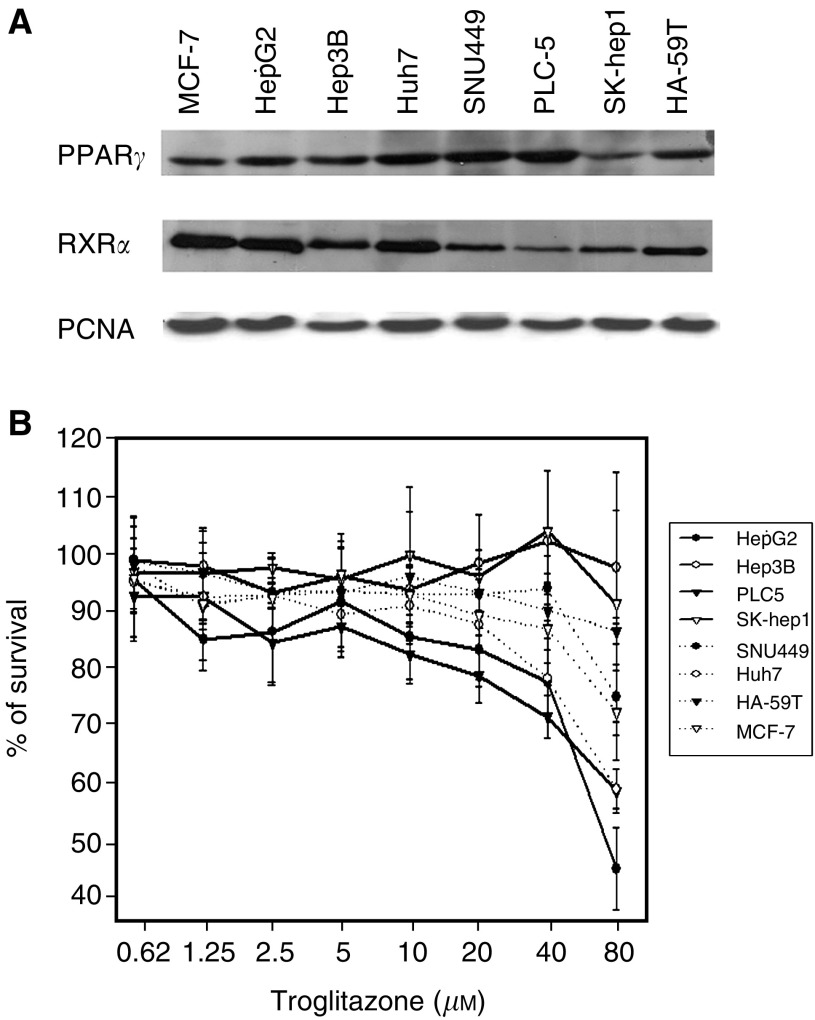
(**A**) Western blot analysis of PPAR*γ* and RXR*α* expression in nuclear protein lysate of HCC cells. All tested HCC cell lines expressed various levels of PPAR*γ* and RXR*α* constitutively. PCNA staining was used as loading control. (**B**) Growth inhibition of HCC cells induced by troglitazone determined by MTT assay. Growth inhibition was noted at concentration more than 20 *μ*M, and the degree of inhibition did not correlate with the expression levels of PPAR*γ* or RXR*α*.

The nuclear expression of PPAR*γ* and RXR*α* and the growth inhibitory effect of troglitazone on HCC cells are shown in Figure 1

. All tested HCC cell lines expressed various levels of PPAR*γ* and RXR*α* constitutively (Figure 1A). Troglitazone, up to 10 *μ*M, had no significant growth inhibitory activity on any of the HCC cell lines. Growth inhibition was noted at concentration more than 20 *μ*M, and the degree of inhibition did not correlate with the expression levels of PPAR*γ* or RXR*α* (Figure 1B).

### Combination activity of troglitazone and 9-*cis* RA

**Figure 2:**
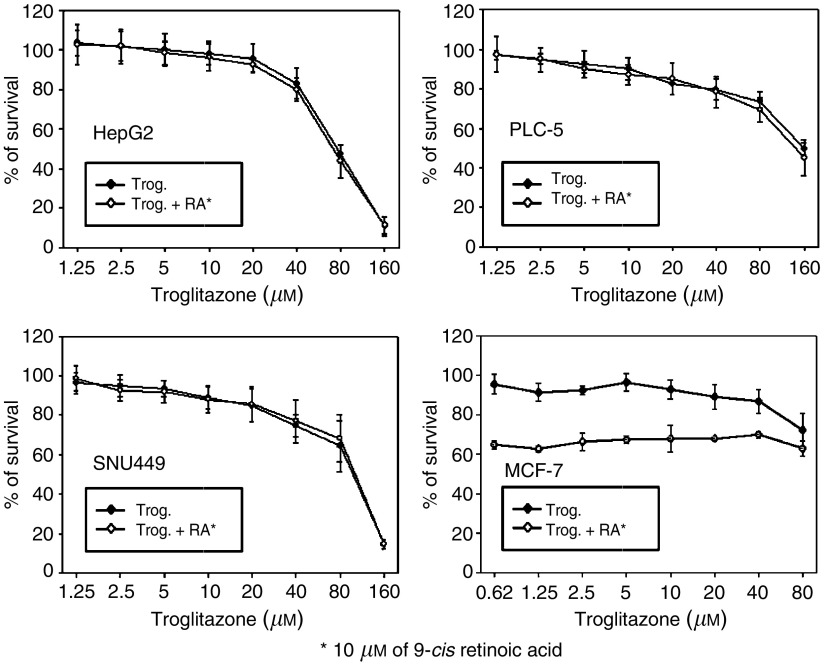
Growth inhibition of HCC cells induced by troglitazone with or without the addition of 10 *μ*M of 9-*cis*-RA. Addition of 9-*cis*-RA did not affect the sensitivity of HCC cells to troglitazone.

Addition of 10 *μ*M of 9-*cis*-RA caused 60% growth inhibition of MCF-7 cells but had no significant growth inhibitory effects on any of the HCC cells and did not affect the sensitivity of HCC cells to troglitazone (Figure 2

).

### Combination activity of troglitazone and anticancer agents

The IC50 of the four cytotoxic agents in three HCC cell lines, with and without the addition of 10 *μ*M of troglitazone, is listed in Table 1

**Table 1:** IC_50_ (*μ*M) of cytotoxic agents with or without troglitazone in HepG2 and PLC cell lines

	**HepG2**	**PLC-5**
Cisplatin	5.1±0.6	52.2±1.5
Cisplatin+T	5.0±1.4	55.5±1.3
Gemcitabine	0.65±0.40	47.3±4.8
Gemcitabine+T	0.87±0.31	43.3±8.4
Paclitaxel	0.95±0.19	6.9±1.5
Paclitaxel+T	0.79±0.21	5.7±1.2
Irinotecan	37.7±8.2	427.5±66.8
Irinotecan+T	36.4±7.3	452.2±122.2

T=troglitazone 10 *μ*M.

The IC50 of individual cell lines was calculated by using a linear regression based on the MTT assay results. The drug concentration corresponding to 50% inhibition of control was designated as IC50.

. The addition of troglitazone did not change the sensitivity of HCC cells toward any of the cytotoxic drugs.

## DISCUSSION

This study indicates that troglitazone does not significantly inhibit the growth of HCC cells at clinically achievable concentrations, and this lack of activity could not be improved by the addition of 9-*cis*-RA. Furthermore, no synergistic effect was found between troglitazone and four representative anticancer agents.

There was no apparent correlation between the degree of growth inhibition by troglitazone and the level of PPAR*γ* or RXR*α* expression in our study. No correlation of the sensitivity of HCC cells to troglitazone with expression levels of PPAR*γ* has been noted (Koga *et al*, 2001; Rumi *et al*, 2001; Okano *et al*, 2002). Similarly, previous studies for the sensitivity of cancer cells to retinoids have demonstrated that the growth inhibitory effects of retinoids did not correlate to the expression levels of different isoforms of retinoic acid receptors and RXRs (van der Leede *et al*, 1993). Other factors, including the levels of ‘free’ RXR*α* that is available for PPAR*γ*-RXR*α* interaction, the content of other nuclear receptors, and the possible interaction among nuclear coactivators and corepressors, may play an important role in determining the sensitivity of cancer cells to the nuclear receptor agonists (Torchia *et al*, 1998).

Although troglitazone and other thiazolidinediones are considered specific PPAR*γ* agonists, several lines of evidence suggest that the growth inhibition of HCC cells induced by high concentrations of troglitazone may occur through PPAR*γ*-independent mechanisms. First, thiazolidinediones induce differentiation of adipose tissue and insulin sensitization at submicromolar levels and these effects are closely related to their binding affinity to PPAR*γ* receptor (Lehmann *et al*, 1995; Goldstein, 2002). On the other hand, concentrations of troglitazone and other thiazolidinediones required to induce significant anticancer effect are usually 10 *μ*M or higher. Besides, no evident relationship between anticancer efficacy and the expression of PPAR*γ* in cancer cells or the binding affinity of thiazolidinediones to PPAR*γ* has been established. Second, addition of RXR*α* agonist may also improve the insulin-sensitising effect of thiazolidinediones by enhancing the formation of PPAR*γ*–RXR*α* heterodimer (Mukherjee *et al*, 1997). This synergistic effect has also been found in liposarcoma and breast cancer cell lines but not in the present study, in spite of the constitutive expression of PPARγ and RXR*α* in the nuclei of HCC cells. Third, inhibition of cell growth by troglitazone through PPAR*γ*-independent mechanisms has also been demonstrated in PPAR*γ*^−/−^ embryonal stem cells (Palakurthi *et al*, 2001). Troglitazone may inactivate the eukaryotic initiation factor 2 (eIF2) and abrogate the expression of G1 cyclins, thus resulting in cell cycle arrest at G1–S transition. Because the concentration of troglitazone needed to induce cell cycle arrest (10 *μ*M or higher) is significantly higher than the clinically achievable serum concentration (2–5 *μ*M), the clinical usefulness of troglitazone alone as an anticancer agent for HCC appears to be limited.

Troglitazone has also been demonstrated to induce cell cycle arrest through increased expression of the cyclin-dependent kinase inhibitors p21^WAF1/Cip1^, p27^Kip1^, and p18^INK4c^. Cell cycle modulation has been intensely investigated as a novel way to induce sensitisation to chemotherapeutic drugs as well as to inhibit cancer cell growth (Senderowicz and Sausville, 2000; Sherr, 2000). However, the concentration of troglitazone necessary to induce this effect is much higher than that achievable clinically. Four major anticancer agents were tested in this study for their effect in combination with troglitazone. The lack of synergistic activity between troglitazone and cytotoxic drugs in this study suggests that troglitazone may not be effective as a biochemical modulator for HCC.

The reason for the lack of efficacy of troglitazone as a biochemical modulator for HCC cells remained undetermined. Most of the HCC cell lines came from patients with surgically resected tumours. Some cell lines, such as PLC5, have been found to have integration of hepatitis B viral DNA into the host genome. The implication of viral DNA integration into host cells on drug sensitivity is not known, although *in vitro* data suggested that expression of hepatitis B viral proteins may increase apoptosis threshold and resistance to cytotoxic agents of cancer cells (Doong *et al*, 1998; Shih *et al*, 2000).

Nevertheless, the PPAR*γ* agonists may have other anticancer effects. It has been demonstrated that PPAR*γ* agonists are potent inhibitors of angiogenesis (Xin *et al*, 1999). Rosiglitazone, another thiazolidinedione derivative, has been shown to inhibit both primary tumour growth and metastasis (Panigrahy *et al*, 2002). The potential mechanisms of action include direct inhibition of endothelial cell proliferation, decrease of vascular endothelial growth factor production by tumour cells, and increased activity of the tissue inhibitor of matrix metalloproteinase (TIMP). Notably, the concentrations of rosiglitazone that had the strongest antiproliferative effect on endothelial cells (0.01–1 *μ*M) are clinically achievable. Therefore, it remains a possibility that new PPAR*γ* agonists with novel antitumour mechanisms that are effective for the treatment of HCC can be developed.

In conclusion, troglitazone, at clinically achievable concentrations, does not appear to be active against HCC cells, either alone or in combination with 9-*cis*-RA or chemotherapeutic agents. Further exploration for new derivatives of PPAR*γ* agonists with better antitumour activity in HCC is needed.

## References

[bib1] Berger J, Bailey P, Biswas C, Cullinan CA, Doebber TW, Hayes NS, Saperstein R, Smith RG, Leibowitz MD (1996) Thiazolidinediones produce a conformational change in peroxisomal proliferator-activated receptor-gamma: binding and activation correlate with antidiabetic actions in db/db mice. Endocrinology 137: 4189–4195 8828476 10.1210/endo.137.10.8828476

[bib2] Chuang SE, Yeh PY, Lu YS, Lai G.M, Liao CM, Gao M, Cheng AL (2002) Basal levels and patterns of anticancer drug-induced activation of nuclear factor *κ*B (NF-*κ*B), and its attenuation by tamoxifen, dexamethasone, and curcumin in carcinoma cells. Biochem Pharmacol 63: 1709–1716 12007574 10.1016/s0006-2952(02)00931-0

[bib3] Corton JC, Anderson SP, Stauber A (2000) Central role of peroxisome proliferator-activated receptors in the action of peroxisome proliferators. Ann Rev Med 40: 491–518 10.1146/annurev.pharmtox.40.1.491 10836145

[bib4] Demetri GD, Fletcher CD, Mueller E, Sarraf P, Naujoks R, Campbell N, Spiegelman BM, Singer S (1999) Induction of solid tumor differentiation by the peroxisome proliferator-activated receptor-*γ* ligand troglitazone in patients with liposarcoma. Proc Natl Acad Sci USA 96: 3951–3956 10097144 10.1073/pnas.96.7.3951 PMC22401

[bib5] Desvergene B, Wahli W (1999) Peroxisome proliferator-activated receptors: nuclear control and metabolism. Endocrinol Rev 20: 649–688 10.1210/edrv.20.5.0380 10529898

[bib6] Doong SL, Lin MH, Tsai MM, Li TR, Chuang SE, Cheng AL (1998) Transactivation of the human MDR1 gene by hepatitis B virus X gene product. J Hepatol 29: 872–878 9875632 10.1016/s0168-8278(98)80113-x

[bib7] Fogh J, Fogh JM, Orfeo (1977) One hundred and twenty-seven cultured human tumor cell lines producing tumors in nude mice. J Natl Cancer Inst 59: 221–225 327080 10.1093/jnci/59.1.221

[bib8] Goldstein BJ (2002) Differentiating members of the thiazolidinedione class: a focus on efficacy. Diabetes Metab Res Rev 18: S16–S22 11921434 10.1002/dmrr.251

[bib9] Gottardis MM, Lamph WW, Shalinsky DR, Wellstein A, Heyman RA (1996) The efficacy of 9-*cis* retinoic acid in experimental models of cancer. Breast Cancer Res Treat 38: 85–96 8825126 10.1007/BF01803787

[bib10] Kersten S, Desvergne B, Wahli W (2000) Roles of PPARs in health and disease. Nature 405: 421–424 10839530 10.1038/35013000

[bib11] Kliewer A, Umesono K, Noonan DJ, Heyman RA, Evans RM (1992) Convergence of 9-*cis* retinoic acid and peroxisome proliferator signaling pathways through heterodimer of formation of their receptors. Nature 358: 771–774 1324435 10.1038/358771a0 PMC6159883

[bib12] Koeffler HP (2003) Peroxisome proliferator-activated receptor *γ* and cancers. Clin Cancer Res 9: 1–9 12538445

[bib13] Koga H, Sakisaka S, Harada M, Takagi T, Hanada S, Taniguchi E, Okuyama T, Rukuda R, Nagasue N, Kinoshita Y (2001) Involvement of p21^WAF1/Cip1^, p27^Kip1^, and p18^INK4c^ in troglitazone-induced cell-cycle arrest in human hepatoma cell lines. Hepatology 33: 1087–1097 11343236 10.1053/jhep.2001.24024

[bib14] Kubota T, Koshizuka K, Williamson EA, Asou H, Said JW, Holden S, Miyoshi I, Koeffler HP (1998) Ligand for peroxisome proliferator-activated receptor *γ* (troglitazone) has potent antitumor effect against human prostate cancer both *in vitro* and *in vivo*. Cancer Res 58: 3344–3352 9699665

[bib15] Lehmann JM, Moore LB, Smith-Oliver TA, Wilkison WO, Willson TM, Kliewer SA (1995) An antidiabetic thiazolidinedione is a high affinity ligand for peroxisome proliferator-activated receptor *γ*. J Biol Chem 270: 12953–12956 7768881 10.1074/jbc.270.22.12953

[bib16] Mehta RG, Williamson E, Patel MK, Koeffler HP (2000) A ligand of peroxisome proliferators-activated receptor *γ*, retinoids, and prevention of preneoplastic mammary lesions. J Natl Cancer Inst 92: 418–423 10699072 10.1093/jnci/92.5.418

[bib17] Mosmann T (1983) Rapid colorimetric assay for cellular growth and survival: application to proliferation and cytotoxic assays. J Immunol Methods 65: 55–63 6606682 10.1016/0022-1759(83)90303-4

[bib18] Mukherjee R, Davies PJ, Crombie DL, Bischoff ED, Cesario RM, Jow L, Hamann LG, Boehm MF, Mondon CE, Nadzan AM, Paterniti JR, Heyman RA (1997) Sensitization of diabetic and obese mice to insulin by retinoid X receptor agonists. Nature 386: 407–410 9121558 10.1038/386407a0

[bib19] Nakabayashi H, Taketa K, Miyano K, Yamane T, Sato J (1982) Growth of human hepatoma cells lines with differentiated functions in chemically defined medium. Cancer Res 42: 3858–3863 6286115

[bib20] Okano H, Shiraki K, Inoue H, Yamanaka T, Deguchi M, Sugimoto K, Sakai T, Ohmori S, Fujikawa K, Murata K, Nakano T (2002) Peroxisome proliferator-activated receptor *γ* augments tumor necrosis factor family-induced apoptosis in hepatocellular carcinoma. Anti-Cancer Drugs 13: 59–65 11914642 10.1097/00001813-200201000-00007

[bib21] Palakurthi SS, Aktas H, Grubissich LM, Mortensen RM, Halperin JA (2001) Anticancer effects of thiazolidinediones are independent of peroxisome proliferator-activated receptor *γ* and mediated by inhibition of translation initiation. Cancer Res 61: 6213–6218 11507074

[bib22] Panigrahy D, Singer S, Shen LQ, Butterfield CE, Freedman DA, Chen EJ, Moses MA, Kilroy S, Duensing S, Fletcher C, Fletcher JA, Hlatky L, Hahnfeldt P, Folkman J, Kaipainen A (2002) PPAR gamma ligands inhibit primary tumor growth and metastasis by inhibiting angiogenesis. J Clin Invest 110: 923–932 12370270 10.1172/JCI15634 PMC151148

[bib23] Park JG, Lee JH, Kang MS, Park KJ, Jeon YM, Lee HJ, Kwon HS, Park HS, Yeo KS, Lee KU et al (1995) Characterization of cell lines established from human hepatocellular carcinoma. Int J Cancer 62: 276–282 7543080 10.1002/ijc.2910620308

[bib24] Rumi MAK, Sato H, Ishihara S, Kawashima K, Hamamoto S, Kazumori H, Okuyama T, Rukuda R, Nagasue N, Kinoshita Y (2001) Peroxisome proliferator-activated receptor *γ* ligand-induced growth inhibition of human hepatocellular carcinoma. Br J Cancer 84: 1640–1647 11401318 10.1054/bjoc.2001.1821 PMC2363681

[bib25] Sarraf P, Mueller E, Jones D, King FJ, DeAngelo DJ, Patridge JB, Holden SA, Chen LB, Singer S, Fletcher C, Spiegelman BM (1998) Differentiation and reversal of malignant changes in colon cancer through PPAR*γ*. Nat Med 4: 1046–1052 9734398 10.1038/2030

[bib26] Senderowicz AM, Sausville EA (2000) Preclinical and clinical development of cyclin-dependent kinase modulators. J Natl Cancer Inst 92: 376–387 10699068 10.1093/jnci/92.5.376

[bib27] Sherr CJ (2000) The Pezcoller lecture: cancer cell cycles revisited. Cancer Res 60: 3689–3695 10919634

[bib28] Shih WL, Kuo ML, Chuang SE, Cheng AL, Doong SL (2000) Hepatitis B virus X protein inhibits transforming growth factor-*β*-induced apoptosis through the activation of phosphatidylinositol 3-kinase pathway. J Biol Chem 275: 25858–25864 10835427 10.1074/jbc.M003578200

[bib29] Spencer CM, Markham A (1997) Troglitazone. Drugs 54: 89–101 9211083 10.2165/00003495-199754010-00010

[bib30] Tafuri SR (1996) Troglitazone enhance differentiation, basal glucose uptake, and Glut1 protein levels in 3T3 adipocytes. Endocrinology 137: 4706–4712 8895337 10.1210/endo.137.11.8895337

[bib31] Tontonoz P, Singer S, Forman BM, Sarraf P, Fletcher JA, Christopher DM, Fletcher CD, Brun RP, Mueller E, Altiok S, Oppenheim H, Evans RM, Spiegelman BM (1997) Terminal differentiation of human liposarcoma cells induced by ligands for peroxisome proliferator-activated receptor gamma and the retinoid X receptor. Proc Natl Acad Sci USA 94: 237–241 8990192 10.1073/pnas.94.1.237 PMC19300

[bib32] Torchia J, Glass C, Rosenfeld MG (1998) Co-activators and co-repressors in the integration of transcriptional responses. Curr Opin Cell Biol 10: 373–383 9640539 10.1016/s0955-0674(98)80014-8

[bib33] Tsibris JCM, Porter KB, Jazayeri A, Tzimas G, Nau H, Huang H, Kuparadze K, Porter GW, O'Brien WF, Spellacy WN (1999) Human uterine leiomyomata express higher levels of peroxisome proliferator activated receptor *γ*, retinoid X receptor *α*, and all-*trans* retinoic acid than myometrium. Cancer Res 59: 5737–5744 10582693

[bib34] van der Leede BM, van den Brink CE, van der Saag PT (1993) Retinoic acid receptor and retinoid X receptor expression in retinoic acid-resistant human tumor cell lines. Mol Carcinog 8: 112–122 7691069 10.1002/mc.2940080208

[bib35] Vanecq J, Latruffe N (1999) Medical significance of peroxisome proliferator-activated receptors. Lancet 354: 141–148 10408502 10.1016/S0140-6736(98)10364-1

[bib36] Willson TM, Lambert MH, Kliewer SA (2001) Peroxisome proliferators-activated *γ* and metabolic disease. Annu Rev Biochem 70: 341–367 11395411 10.1146/annurev.biochem.70.1.341

[bib37] Wuu KD, Wuu SW, Hu CP, Chang CM (1990) A human hepatocellular carcinoma cell line with multiple copies of structurally normal chromosomes. J Formos Med Assoc 89: 1–5 1973703

[bib38] Xin X, Yang S, Kowalski J, Gerritsen ME (1999) Peroxisome proliferator-activated receptor gamma ligands are potent inhibitors of angiogenesis *in vitro* and *in vivo*. J Biol Chem 274: 9116–9121 10085162 10.1074/jbc.274.13.9116

[bib39] Yoshizawa K, Cioca DP, Kawa S, Tanaka E, Kiyosawa K (2002) Peroxisome proliferator-activated receptor *γ* ligand troglitazone induces cell cycle arrest and apoptosis of hepatocellular carcinoma cell lines. Cancer 95: 2243–2251 12412180 10.1002/cncr.10906

